# Criterion Validation of Tau PET Staging Schemes in Relation to Cognitive Outcomes

**DOI:** 10.3233/JAD-230512

**Published:** 2023-10-24

**Authors:** Dustin B. Hammers, Joshua H. Lin, Angelina J. Polsinelli, Paige E. Logan, Shannon L. Risacher, Adam J. Schwarz, Liana G. Apostolova, for the Alzheimer’s Disease Neuroimaging Initiative

**Affiliations:** a Department of Neurology, Indiana University School of Medicine, Indianapolis, IN, USA; b Department of Radiology and Imaging Sciences, Indiana University School of Medicine, Indianapolis, IN, USA; c Takeda Pharmaceuticals Ltd., Cambridge, MA, USA

**Keywords:** Alzheimer’s disease, amyloid, learning, memory, mild cognitive impairment, tau

## Abstract

**Background:**

Utilization of NIA-AA Research Framework requires dichotomization of tau pathology. However, due to the novelty of tau-PET imaging, there is no consensus on methods to categorize scans into “positive” or “negative” (T+ or T–). In response, some tau topographical pathologic staging schemes have been developed.

**Objective:**

The aim of the current study is to establish criterion validity to support these recently-developed staging schemes.

**Methods:**

Tau-PET data from 465 participants from the Alzheimer’s Disease Neuroimaging Initiative (aged 55 to 90) were classified as T+ or T– using decision rules for the Temporal-Occipital Classification (TOC), Simplified TOC (STOC), and Lobar Classification (LC) tau pathologic schemes of Schwarz, and Chen staging scheme. Subsequent dichotomization was analyzed in comparison to memory and learning slope performances, and diagnostic accuracy using actuarial diagnostic methods.

**Results:**

Tau positivity was associated with worse cognitive performance across all staging schemes. Cognitive measures were nearly all categorized as having “fair” sensitivity at classifying tau status using TOC, STOC, and LC schemes. Results were comparable between Schwarz schemes, though ease of use and better data fit preferred the STOC and LC schemes. While some evidence was supportive for Chen’s scheme, validity lagged behind others—likely due to elevated false positive rates.

**Conclusions:**

Tau-PET staging schemes appear to be valuable for Alzheimer’s disease diagnosis, tracking, and screening for clinical trials. Their validation provides support as options for tau pathologic dichotomization, as necessary for use of NIA-AA Research Framework. Future research should consider other staging schemes and validation with other outcome benchmarks.

## INTRODUCTION

While the etiology of Alzheimer’s disease (AD) has long been associated with the accumulation of amyloid-β (Aβ) plaques and neurofibrillary tau tangles [1], only within the past couple decades have imaging substrates been developed to identify deposition of these AD biomarkers *in vivo* using positron emission tomography (PET). This increased identification has ushered in a new phase of AD detection, whereby biomarker status has become integral in diagnosis and management of AD [2]. These trends were reflected in the recent creation of the National Institute of Aging–Alzheimer’s Association (NIA-AA) “ATN” Research Framework [3], which defines AD as a biological construct by the presence of Aβ deposition (A), tau deposition (T), and neurodegeneration (N; as evidenced by atrophy on magnetic resonance imaging). This framework consequently requires the dichotomization of AD biomarkers for its proper use. Unlike Aβ [4], and to some extent, neurodegenerative changes [5, 6], however, due to the relative novelty of tau-PET imaging and the challenges associated with standardizing tau topography, tracers, and signal thresholds [7], there is no consensus yet on methods to categorize tau-PET scans into “positive” or “negative”.

In response to this need for dichotomization, a handful of staging schemes have been developed to characterize tau positivity (T+) versus tau negativity (T–). Each of these topographical staging schemes consider the hierarchical spreading of tau pathology by examining the quantity and regional temporal involvement of tau deposition in participants undergoing PET imaging. These quantity x temporal location staging schemes result in profiles of tau deposition, which generally follow a consistent pattern of spread from the inferior and lateral temporal cortices, to parietal and frontal cortices, followed by primary visual cortices [8, 9].

The first set of topographical pathologic staging schemes examined in this study was created by Schwarz and colleagues [10, 11] based on ^18^F-Flortaucipir PET imaging. Two of these schemes (*Temporal-Occipital Classification* [TOC] and the *Simplified Temporal-Occipital Classification* [STOC]) were developed using algorithms based on regions of interest (ROIs) in the anterior temporal and occipital lobes, and follow classification rules consistent with Braak and colleagues’ [9] neuropathologic staging scheme. STOC uses a smaller number of larger ROIs and less complicated staging rules than TOC. A third scheme (*Lobar Classification* [LC]), using fewer even larger ROIs reflects a greater proportion of the overall cortical gray matter by incorporating whole-lobe temporal, parietal, and frontal cortices. Using a sample size of 98 participants across the AD spectrum, Schwarz and colleagues [10] found that all three schemes displayed a high concordance between tau positivity and Aβ positivity (82%–96%), with only 35% to 41% of Aβ positive participants being classified as tau negative. The schemes also reflected similar rates of T+/T– with each other (90%–94% concordance).

A fourth topographic staging scheme using ^18^F-Flortaucipir examined here was recently created by Chen and colleagues [12], which appears to be advanced from initial work by earlier models [13, 14]. Similar to the Schwarz models, the *Chen Classification* (Chen) scheme used Braak stages of neuropathology [9] to create a staging scheme that reflected the intensity and temporality of tau deposition in AD, based on 734 participants along the AD spectrum in the Alzheimer’s Disease Neuroimaging Initiative (ADNI; [15]). As such, three composite ROIs (Braak I/II, Braak III/IV, and Braak V/VI) were incorporated into the model, corresponding to transentorhinal/hippocampal, limbic, and neocortical regions, respectively. Unlike Schwarz models, however, the Chen scheme validated its staging system using both Aβ and longitudinal cognitive trajectories. They observed that greater severity of tau-PET staging resulted in higher rates of cognitive decline, clinical progression, cerebrospinal fluid AD biomarker accumulation, and amyloid-PET deposition.

The current study sought to advance the use of the NIA-AA Research Framework in future research by establishing criterion validity for these different tau pathologic staging schemes in a sample of participants across the AD continuum. Direct comparison of these four schemes is also needed as each scheme differs in terms of size and number of ROIs, complexity of staging rules and number of stages, and standardized uptake value ratio (SUVR) thresholds used for dichotomization. Traditional measures of episodic memory have been used in the current study given their known association with AD pathology [16], and learning slopes have also been incorporated owing to their association with both episodic-memory-related and working memory/attention-related aspects of cognition [17], along with hippocampal [18], ventrolateral prefrontal [17], and dorsolateral prefrontal atrophy [19]. Including both traditional and process-related scores permits potential validation with a wider range of cognitive benchmarks than traditional memory measures alone. It is hypothesized that all four staging schemes would display concordance with both traditional measures of memory and process-related learning slope metrics, but that schemes incorporating a greater number of ROIs and stages, and more detailed scoring rules (e.g., TOC and Chen schemes), would reflect greater sensitivity to cognitive deficit and better diagnostic accuracy. By establishing concordance between neuroanatomical and clinical outcome markers of AD in these schemes, we hope to enhance their utility in identifying tau-PET positivity and increase confidence in their application within the ATN framework. Given the associations between cerebral tau deposition and early memory changes in AD [20], such validation may similarly inform predictive capacity of future cognitive decline.

Please note that because of the high number of abbreviations used frequently throughout the manuscript, a list of abbreviations is included in Supplementary Table 1.

## METHODS

We obtained participant data for the current study from the ADNI multi-center longitudinal study (http://adni.loni.usc.edu). ADNI [21] was launched in 2003 and is a public-private partnership with scientific goals of examining progression of mild cognitive impairment (MCI) and early AD dementia using magnetic resonance imaging, PET, other biological markers, and clinical and neuropsychological assessments. See http://www.adni-info.org for up-to-date information. Written informed consent was obtained from study participants or authorized representatives, and Institutional Review Board approval has been obtained for each multi-center site within the ADNI consortium. All conducted research is in accord with the Helsinki Declaration of 1975.

**Fig. 1:**
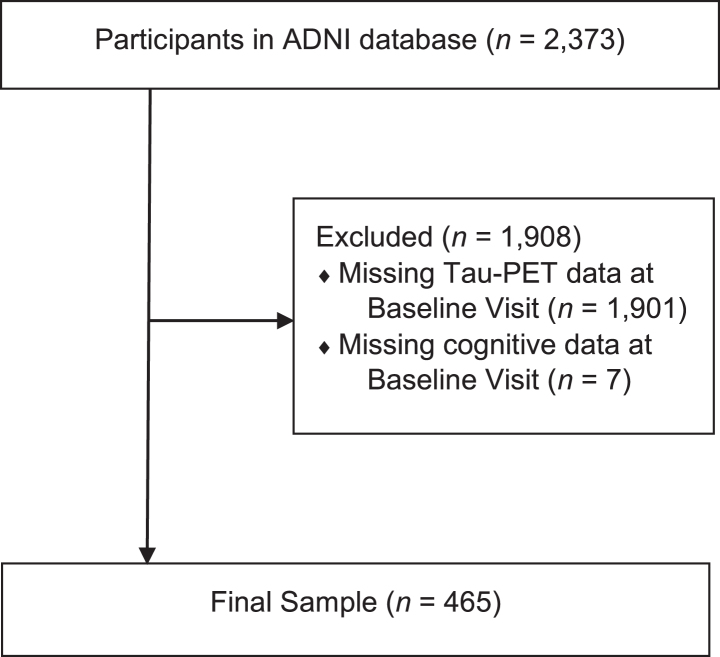
Flow diagram of participants recruited into the current study from the total sample of ADNI participants.

Data were available for 2,373 ADNI participants enrolled in various ADNI protocols [15, 22–24] as of April 26, 2021. Participant data collection began on August 23, 2005, with enrolled participants being followed cognitively for up to 180 months. Tau-PET investigation was initiated in 2017. Inclusion criteria for these ADNI protocols included: being between the ages of 55 to 90 at baseline; the presence of a reliable study partner; having≥6 years of education; absence of significant head trauma, depression, or neurologic disease; stability on permitted medications; and fluency in either English or Spanish. For the current study, 1,901 participants were excluded for not having tau-PET data from their baseline visit, and 7 participants were excluded for having missing baseline cognitive data. Consequently, 465 participants were included in the current study across all disease stages. Please see Fig. 1 for a schematic representation of the current study’s participant utilization from the ADNI.

### Tau-PET scan preprocessing

All participants in the current study underwent tau-PET imaging using ^18^F-Flortaucipir, as per standard ADNI protocols [22, 23]. Please see ADNI protocols for greater details about Tau-PET methods. Pre-processed ADNI ^18^F-Flortaucipir 80–100-min smoothed static scans were downloaded (http://adni.loni.usc.edu) and processed using standard techniques in Statistical Parametric Mapping-12. Briefly, static Tau-PET scans were rigidly co-registered to the closest in-time structural magnetic resonance imaging scan. Then, the structural magnetic resonance imaging scans were segmented using voxel-based morphometry in Statistical Parametric Mapping-12 to generate matrices describing needed transformations to normalize to standard Montreal Neurologic Institute space. Next, the matrices were applied to the co-registered tau-PET images to normalize them to standard Montreal Neurologic Institute space. Finally, SUVR images were generated by intensity normalization to a cerebellar crus ROI.

### Tau-PET topographical staging schemes

The classification of tau positivity from the baseline visit was conducted using a series of tau-PET topographical staging schemes for AD. Briefly, mean ^18^F-Flortaucipir SUVR was extracted with grey matter masking from the ROIs detailed below and used to generate tau stage classification for all participants. For the interested reader, further details about the methods used by the original authors for deriving SUVR cutoffs can be observed in the Supplementary Material. The criterion and resulting classifications for the staging schemes adhered to developers’ protocols, as follows:

Schwarz staging schemes: Schwarz and colleagues’ [10] work characterized three separate pathologic staging schemes based on pre-defined patterns of tau-burden.

1. The TOC model used small ROIs in the anterior temporal and occipital lobes, along with classification rules designed for consistency with Braak and colleagues’ [9] six-stage operationalized neuropathologic staging scheme. Specifically, the TOC scheme incorporated the following brain regions based on the associated SUVR cutoff (in parentheses): hippocampus (SUVR threshold of≥1.222), transentorhinal cortex (≥1.310), fusiform gyrus (≥1.352), middle temporal gyrus (≥1.296), superior temporal gyrus (≥1.219), extrastriate visual cortex (≥1.308), and primary visual cortex (≥1.268). Resultant patterns of positivity led to a Pathologic Staging score ranging from 0–6, with scores of 0–3 being considered T–, and scores of 4–6 being considered T+ [10]. Specifically, T+ corresponds to PET positivity in the middle temporal gyrus and extrastriate visual cortex, whereas scans with regional positivity restricted to medial temporal regions (transentorhinal cortex, hippocampus, and fusiform gyrus) were considered T–.
2. The STOC model used larger ROIs associated with fewer regions within the anterior temporal and occipital lobes, along with less complex decision rules. Specifically, the STOC scheme incorporated the following brain regions based on the associated SUVR cutoff (in parentheses): medial temporal lobe (SUVR threshold of≥1.222), lateral temporal lobe (≥1.306), superior temporal gyrus (≥1.255), and primary visual cortex (≥1.310). Resultant patterns of positivity led to a Pathologic Staging score ranging from 0–4, with scores of 0–1 being considered T–, and scores of 2–4 being considered T+ [10]. Consistent with the TOC decision rule, T+ corresponds to regional positivity in the lateral temporal lobe, whereas scans with regional positivity restricted to the medial temporal lobe ROI were considered T–.
3. The LC model used the largest and fewest ROIs, and the least complex decision rules, of the Schwarz schemes. Specifically, the LC scheme incorporated whole-lobe data based on the associated SUVR cutoff (in parentheses): temporal lobe (SUVR threshold of≥1.263), parietal lobe (≥1.297), frontal lobe (≥1.290). Resultant patterns of positivity led to a Pathologic Staging score ranging from 0–, with scores of 0 being considered T–, and scores of 1–3 being considered T+ [10]. In this scheme, since there is only a single ROI for the entire temporal lobe, T– scans correspond to below-threshold SUVR values in all ROIs.

Chen staging scheme: Pre-defined patterns of tau-burden were identified according to ROIs localized around transentorhinal/hippocampal, limbic, and neocortical regions, which mapped onto Braak stages I/II, III/IV, and V/IV, respectively [12]. Stage 4 was assigned to participants with Braak V/VI SUVR > 1.873. Subthreshold participants with Braak III/IV SUVR < 1.873 and > 1.523 were assigned to Stage 3. Subthreshold participants with Braak III/IV SUVR < 1.523 and > 1.307 were assigned to Stage 2. Subthreshold participants with Braak I/II SUVR > 1.130 were assigned to stage 1. All remaining participants were assigned to stage 0. Resultant patterns of positivity led to a Pathologic Staging score ranging from 0–4, with a score of 0 being considered T–, and scores of 1–4 being considered T+ [12]. Consequently, T+ corresponds to PET positivity anywhere in the transentorhinal, limbic, and neocortical regions, and T– scans correspond to below-threshold SUVR values inall ROIs.

### Actuarial diagnostic classification

For further characterization of the AD biomarker status groups, participants were classified into diagnostic groups (cognitively normal, MCI, or dementia due to AD). Because of recent critique of ADNI’s diagnostic classification [25], a modified version of Jak/Bondi and colleagues’ [26, 27] actuarial model of diagnosis for MCI was used in the current study. Of note, as the modified Jak/Bondi criteria is only used to discern participants with MCI versus normal cognition, ADNI diagnostic classification of participants with AD dementia were unaltered. For participants with an ADNI diagnosis of either MCI or normal cognition, age-, education-, and sex-adjusted normative scores were generated using published normative data from the National Alzheimer’s Coordinating Center neuropsychological battery [28, 29]. Normative scores were generated for the following measures and domains: Logical Memory I and II (“Story A”) from the Wechsler Memory Scale – Revised [30] for the memory domain, Trail-Making Test Parts A and B [31] for the speed/executive functioning domain, and Category Fluency – Animals [32], and Multi-Lingual Naming Test [33] for the language domain. Participants were classified as having an actuarial diagnosis of MCI if they possessed an ADNI diagnosis of normal cognition or MCI and any of the following criteria were met: the presence of 1) impaired scores (>1 *SD* below the normative mean) on both measures within at least one cognitive domain (i.e., memory, speed/executive function, or language); 2) one impaired score (>1 *SD* below the normative mean) in each of the three cognitive domains; or 3) a score on the Functional Activity Questionnaire [34] ≥6. Note that although Bondi et al. [26] initially used a Functional Activity Questionnaire cutoff of 9, subsequent research from their group modified the cutoff to≥6 [35, 36]. If participants possessed an ADNI diagnosis of normal cognition or MCI and no actuarial criteria were met, then the participants were classified as being cognitivelyintact.

To ensure validity of these actuarial diagnoses, cognitive performance on select variables, hippocampal volumes, and Aβ positivity were examined between diagnostic groups. As seen in Supplementary Table 2, the AD dementia group performed worse than the MCI group (*p*s < 0.001), which performed worse than the cognitively normal group (ps < 0.001) on the Montreal Cognitive Assessment [37], Clinical Dementia Rating Scale [38] – Sum of Boxes, Alzheimer’s Disease Assessment Scale – Cognitive Subscale (ADAS-Cog) [39], Rey Auditory Verbal Learning Test (RAVLT) Immediate Recall [40], and RAVLT Delayed Recall. Similarly, bilateral hippocampal volumes (after controlling for intracranial volume) were significantly smaller for the AD dementia group than the MCI group (*p* < 0.001), which were smaller than those in the cognitively normal group (*p* < 0.001). Finally, the presence of Aβ deposition was significantly different between actuarial diagnostic groups, with rates of Aβ positivity being greater for the AD dementia group (90%) than the MCI group (65%), which were greater than those in the cognitively normal group (45%;ps < 0.001).

### Procedure

All participants underwent an extensive clinical and neuropsychological battery at a baseline visit upon their enrollment in ADNI. For the current study, the relevant neuropsychological measures used were as follows:

• RAVLT is a verbal memory task with 15 words learned across 5 trials, with the number of correct words summed for the Total (or Immediate) Recall score (range = 0–75). The Delayed Recall score is the number of correct words recalled after a 30-min delay (range = 0–15). All RAVLT scores reflect raw scores, with higher values indicating better performance.
• Logical Memory I and II from the Wechsler Memory Scale – Revised are immediate and delayed (20–30 min) memory measures for a verbally presented short story. Only “Story A” was provided to participants based on ADNI-3 protocol, therefore the range of scores for Logical Memory I and II is both 0–23. All values reflect raw scores, with higher values indicating better performance.
• ADAS-Cog comprises 13 subtests pertaining to learning and memory, language production and comprehension, constructional praxis, ideational praxis, orientation, and executive skills. The Total Score ranges from 0–85, with higher scores indicating worse performance. For the present study we focused on two subtests. The Word Recall subtest (officially titled “Question 1” of the ADAS-Cog) is a verbal list-learning task of 10 words learned over 3 trials. Words from this list cannot be easily clustered into semantic categories. The Delayed Recall subtest (officially titled “Question 4” of the ADAS-Cog) is the recall of those words after a 10-min delay. For the purpose of the current study, modifications to test developer’s scoring procedures were undertaken for consistency with all other memory measures in the study (i.e., higher values reflecting better memory performance). Specifically, Immediate Recall in the current study was the number of correct words identified across trials (range = 0–30), and Delayed Recall was the number of correct words recalled after delay (range = 0–10).

Additional neuropsychological test measures used in ADNI are common to most dementia clinicians and researchers, therefore they will not be described here. Readers are referred to ADNI protocols [15, 22–24] for neuropsychological test descriptions and psychometric properties. Additional measures include American National Adult Reading Test [41], Mini-Mental State Examination [42], Montreal Cognitive Assessment, Clinical Dementia Rating Scale – Sum of Boxes, Functional Activity Questionnaire, and the 15-item Geriatric Depression Scale [43]. Higher scores indicated better performance for American National Adult Reading Test, Mini-Mental State Examination, and Montreal Cognitive Assessment. Lower scores indicated better performance for Clinical Dementia Rating Scale – Sum of Boxes, Functional Activity Questionnaire, and Geriatric Depression Scale (cutoff for depression > 5).

### Calculation of learning slopes

Learning slopes were calculated from the Immediate Recall subtest of the RAVLT and Word Recall subtest of the ADAS-Cog. Specifically, the Raw Learning Score (RLS) was computed as the highest performance (on Trials 2 through the Final Trial) minus Trial 1. The Learning Ratio (LR) [44] was represented as a proportion: the RLS score in the numerator, and the total points available for a trial minus Trial 1 in the denominator. Please note that the “Total Points Available for a Trial” for RAVLT is 15 and Word Recall subtest is 10. Learning Over Trials (LOT) score was computed as the total information learned (sum of Trials 1 through the Final Trial) minus the weighted information learned by Trial 1 (value of Trial 1 multiplied by the number of trials presented). There were five trials presented for the RAVLT, and three trials presented for Word Recall (modified from [45]). The formulas for RLS, LOT, and LR derived from the RAVLT and Word Recall subtest of the ADAS-Cog are as follows:

$$\begin{array}{cc} & \;\;\;\mathrm{RLS}=\left(\mathrm{Highest}\mathrm{performance}\mathrm{on}\mathrm{Trials}2\mathrm{through}\mathrm{Final}\mathrm{Trial}–\mathrm{Trial}1\mathrm{performance}\right)\\ & \;\;\;\mathrm{LR}=\frac{\left(\mathrm{Highest}\mathrm{performance}\mathrm{on}\mathrm{Trials}2\mathrm{through}\mathrm{Final}\mathrm{Trial}–\mathrm{Trial}1\mathrm{performance}\right)}{\left(\mathrm{Total}\mathrm{Points}\mathrm{Available}\mathrm{for}a\mathrm{Trial}–\mathrm{Trial}1\mathrm{performance}\right)}\\ & \;\;\;\mathrm{LOT}=\left(\mathrm{Sum}\mathrm{of}\mathrm{Trials}1\mathrm{through}\mathrm{Final}\mathrm{Trial})–(\mathrm{Number}\mathrm{of}\mathrm{Trials}*\mathrm{Trial}1\mathrm{performance}\right) \end{array}$$

### Data analysis

For demographic comparisons and to determine the appropriateness of covariates, independent samples *t* tests were calculated between tau status groups (T–, T+) for continuous demographic variables (e.g., age, education, verbal intellect), and *Chi square* analyses were calculated for categorical demographic variables (e.g., sex and ethnicity). Relevant demographic variables were included as covariates in the subsequent analyses.

For the criterion validity primary analyses, multivariate analysis of covariance was conducted comparing tau biomarker status groups on cognitive performance, using all four tau pathologic staging methodologies. Separate multivariate analysis of covariance were conducted for immediate and delayed memory scores (RAVLT, Logical Memory, ADAS-Cog Word Recall), and learning slope performances (LR, RLS, LOT) derived from the RAVLT and ADAS-Cog Word Recall subtests. Subsequent one-way analyses of covariance were conducted for differences in individual cognitive measures within the omnibus test.

For consideration of test operating characteristics for immediate and delayed memory, and learning slope metrics, receiver operating characteristic area under the curve (ROC-AUC) analyses were conducted between participants in the T– and the T+ groups separately for each pathologic staging scheme. For the interpretation of ROC-AUC values, the current study followed guidelines suggested by Hosmer and colleagues [46] of ROC-AUC values < 0.600 being a “failure”, values between 0.600 and 0.699 being “poor”, values between 0.700 and 0.799 being “fair”, values between 0.800 and 0.899 being “good”, and values 0.900 or greater being “excellent”. Cut scores for cognitive performances were determined based on optimal sensitivity and specificity for detecting the presence of tau pathology.

Finally, diagnostic accuracy metrics (e.g., false positive rate, positive predictive power, negative predictive power) for each pathologic staging scheme were examined by comparing tau positivity rates relative to actuarial diagnoses of cognitively normal and cognitively impaired (either AD dementia or MCI).

Measures of effect size were expressed as Cohen’s d (*t* tests, multivariate analysis of covariance/analysis of covariance) values and Phi coefficients (*χ*^2^). Additionally, comparisons between AUC values were examined using 95% compatibility intervals (CIs). To protect against multiple comparisons, a two-tailed alpha level was set at.01 for all analyses.

## RESULTS

### Demographics

The sample was composed of 465 participants who underwent tau-PET from ADNI (Table 1). The mean age of the sample was 70.9 (SD = 7.1; range 55–90) years old, averaging 16.5 (SD = 2.3; range 10–20) years of education. There was a slight female predominance (55.3% female), with most participants being Caucasian (84.3%). Mean intellect at baseline according to American National Adult Reading Test Verbal Intellect was estimated to be high average (*M* = 118.6, SD = 9.8, range 85–131). The mean Montreal Cognitive Assessment performance for the sample was 24.3 (SD = 4.3, range 7–30), and the mean Clinical Dementia Rating Scale – Sum of Boxes was 1.0 (SD = 1.6, range 0–10). Overall self-reported depression on the 15-item Geriatric Depression Scale was low (*M* = 1.11, SD = 1.4).

**Table 1:** Demographic variables for the biomarker status groups and total sample

Variable	Total	TOC		STOC		LC		Chen	
	Sample	T–	T+	T–	T+	T–	T+	T–	T+
N	465	363	102	341	124	356	109	236	229
Age, y ^1^	70.9 (7.1)	70.5 **^**1**^** (7.0)	72.6 (6.8)	70.5 **^**1**^** (6.9)	72.0 (7.2)	70.6 (6.9)	71.8 (7.1)	69.9 **^**1**^** (6.9)	72.0 (7.0)
Education, y	16.5 (2.3)	16.5 (2.3)	16.5 (2.5)	16.5 (2.3)	16.4 (2.5)	16.6 (2.3)	16.3 (2.5)	16.4 (2.3)	16.6 (2.4)
Sex (% female)	55.3%	57.6%	47.1%	56.9%	50.8%	57.3%	48.6%	58.9%	51.5%
Race (% Caucasian)	84.3%	84.8%	82.4%	84.2%	84.7%	84.3%	84.4%	83.5%	85.2%
MoCA	24.3 (4.3)	25.2 (3.6)	21.1 (5.0)	25.2 (3.5)	21.6 (5.2)	25.2 (3.5)	21.2 (5.1)	25.4 (3.4)	23.1 (4.8)
CDR-SB	1.0 (1.6)	0.7(1.3)	2.1 (2.0)	0.6 (1.2)	1.9 (2.0)	0.7 (1.3)	1.9 (2.0)	0.5 (1.1)	1.4 (1.9)
ADAS-Cog Total Score	16.7 (8.0)	14.7 (6.2)	24.0 (9.3)	14.7 (6.0)	22.7 (9.7)	14.7 (6.2)	23.5 (9.4)	14.0 (5.6)	19.6 (9.0)
FAQ	2.5 (5.2)	1.4 (3.7)	6.5 (7.4)	1.3 (3.6)	5.8 (7.0)	1.4 (3.8)	6.1 (7.1)	1.2 (3.4)	3.9 (6.3)
AMNART Verbal Intelligence	118.6 (9.8)	118.9 (9.8)	117.7 (9.6)	119.0 (9.7)	117.6 (9.9)	119.1 (9.7)	116.9 (9.8)	118.7 (9.7)	118.5 (9.9)
Geriatric Depression Scale	1.1 (1.4)	1.0 (1.3)	1.5 (1.7)	1.1 (1.4)	1.3 (1.5)	1.1 (1.4)	1.4 (1.5)	1.0 (1.3)	1.3 (1.5)
Amyloid status (% positive) ^1^	54%	45% **^**1**^**	87%	43% **^**1**^**	86%	44% **^**1**^**	86%	41% **^**1**^**	68%

TOC, Temporal-Occipital Classification; STOC, Simplified Temporal-Occipital Classification; LC, Lobar Classification Scheme; Chen, Chen Classification; T-, Tau negative; T+, Tau positive; CDR, Clinical Dementia Rating Scale – Global; CDR-SB, Clinical Dementia Rating Scale – Sum of Boxes; MMSE, Mini-Mental State Examination; MoCA, Montreal Cognitive Assessment; ADAS-Cog, Alzheimer’s Disease Assessment Scale – Cognitive Subscale; FAQ, Functional Activities Questionnaire; AMNART, North American Adult Reading Test. All scores are raw scores, and all values are Mean (SD) unless listed otherwise. 1 Denotes significant difference between T- and T+ groups.

The results of classification by the TOC staging scheme led to 363 participants being categorized as T– and 102 as T+. STOC staging led to 341 participants categorized as T– and 124 as T+. LC staging led to 356 participants categorized as T– and 109 as T+. Finally, Chen staging led to 236 participants categorized as T– and 229 categorized as T+. When comparing demographic differences across T+/– groups, Table 1 shows that the T+ group was consistently older than the T– group across pathologic staging schemes (TOC: t(463) = –2.69, *p* = 0.007, *d* = –0.30; STOC: t(463) = –2.08, *p* = 0.04, *d* = –0.22; LC: t(463) = –1.50, *p* = 0.13, *d* = –0.16; and Chen: t(463) = –3.21, *p* = 0.001, *d* = –0.30). No differences were observed for education, sex, or ethnicity (ps > 0.05). For each staging scheme, the proportion of Aβ positive participants were higher in the T+ group than the T– group (*p*s < 0.001, *Phi* = 0.27 to 0.38).

### Criterion validity analyses

**Fig. 2:**
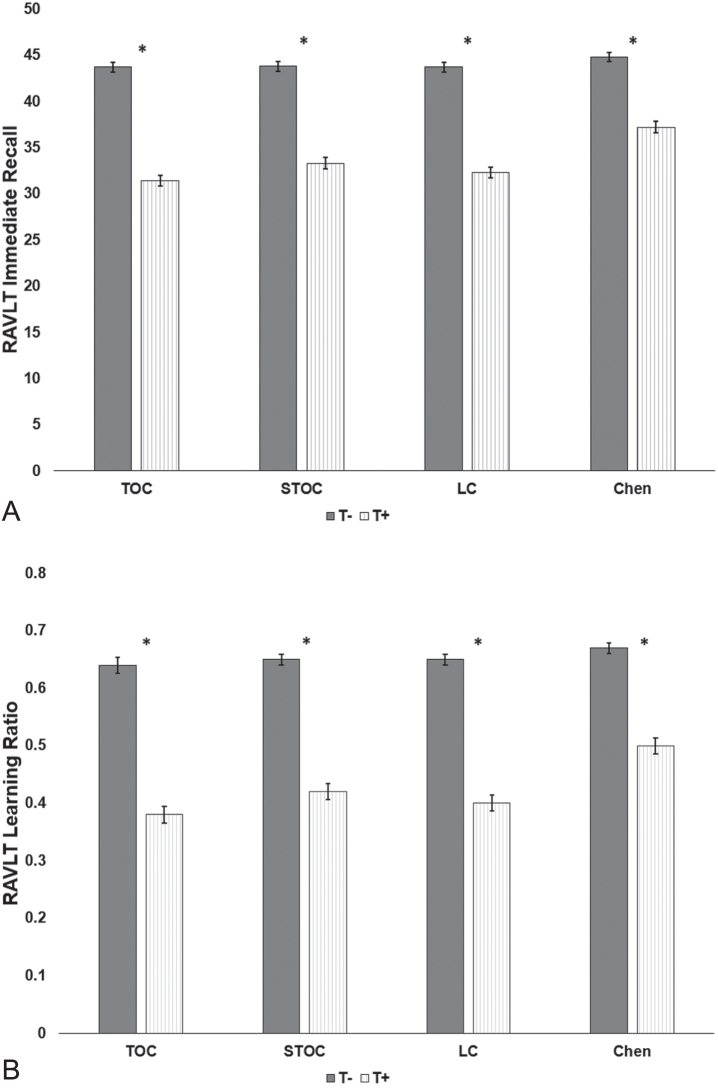
Comparison of performances on the Rey Auditory Verbal Learning Test (RAVLT) Immediate Recall (A) and Learning Ratio (B) variables between for the Tau+and Tau – groups for each of the pathologic staging schemes. TOC, Temporal-Occipital Classification; STOC, Simplified Temporal-Occipital Classification; LC, Lobar Classification Scheme; Chen, Chen Classification; T–, Tau negative; T+, Tau positive. *T+ versus T– comparisons significant, *p* < 0.001.

When comparing learning and memory scores between the T+/– groups (Table 2 and Fig. 2), significant differences were observed across pathologic staging schemes after controlling for age (*Wilk*’*s Lambda* = 0.80 and *Cohen*’*s d* = 1.02 for TOC, *Wilk*’*s Lambda* = 0.80 and *Cohen*’*s d* = 1.01 for STOC, *Wilk*’*s Lambda* = 0.79 and *Cohen*’*s d* = 1.05 for LC, and *Wilk*’*s Lambda* = 0.87 and *Cohen*’*s d* = 0.78 for Chen). Group differences existed across staging schemes for each of the Immediate and Delayed Recall measures (RAVLT, Logical Memory, and Word Recall; *p*s < 0.001). Specifically, Table 3 indicates that the magnitudes of effect for the TOC, STOC, and LC schemes were large (*Cohen*’*s d*s = 0.71 to 0.97 for TOC, *Cohen*’*s d*s = 0.66 to 0.94 for STOC, and *Cohen*’*s d*s = 0.71 to 1.01 for LC; [47]), and the magnitude of effect for the Chen scheme was medium to large (*Cohen*’*s d*s = 0.51 to 0.70). When examining 95% *CI*s, the Chen scheme – with the exception of RAVLT Delayed Memory – consistently failed to overlap with the midpoint of the 95% *CI*s for the TOC, STOC and LC values, suggesting that the magnitude of effect was smaller for that scheme relative to the others [48].

**Table 2:** Learning and memory variables for the Tau+and Tau – groups for each of the pathologic staging schemes

Variable	Total	TOC		STOC		LC		Chen	
	Sample	T–	T+	T–	T+	T–	T+	T–	T+
Learning and Memory
RAVLT Immediate Recall	41.1 (12.6)	43.7 (11.5)	31.4* (12.1)	43.8 (11.3)	33.3* (12.9)	43.7 (11.5)	32.3* (12.3)	44.8 (11.3)	37.2* (12.8)
RAVLT Delayed Recall	6.1 (4.6)	7.0 (4.5)	2.9* (3.7)	7.0 (4.4)	3.6* (4.3)	7.0 (4.5)	3.1* (3.8)	7.5 (4.4)	4.7* (4.4)
Logical Memory Immediate Recall	11.6 (4.9)	12.5 (4.4)	8.4* (5.1)	12.6 (4.4)	8.6* (5.0)	12.5 (4.4)	8.5* (5.2)	12.8 (4.3)	10.3* (5.1)
Logical Memory Delayed Recall	10.0 (5.3)	11.0 (4.8)	6.1* (5.4)	11.2 (4.7)	6.4* (5.3)	11.0 (4.8)	6.4* (5.4)	11.6 (4.6)	8.3* (5.5)
Word Recall Immediate Recall	18.9 (5.4)	20.2 (4.6)	15.1* (5.3)	20.2 (4.6)	15.9* (5.6)	20.2 (4.5)	15.2* (5.5)	20.8 (4.3)	17.5* (5.5)
Word Recall Delayed Recall	6.1 (2.6)	6.7 (2.3)	3.9* (2.5)	6.7 (2.2)	4.2* (2.6)	6.7 (2.2)	4.0* (2.6)	7.0 (2.1)	5.2* (2.7)
Learning Slope
RAVLT LR	0.59 (0.3)	0.64 (0.3)	0.38* (0.3)	0.65 (0.2)	0.42* (0.3)	0.65 (0.2)	0.40* (0.3)	0.67 (0.2)	0.50* (0.3)
RAVLT RLS	5.7 (2.6)	6.1 (2.5)	4.0* (2.3)	6.2 (2.4)	4.2* (2.7)	6.2 (2.4)	4.0* (2.5)	6.4 (2.4)	4.9* (2.6)
RAVLT LOT	15.9 (8.7)	17.5 (8.3)	10.3* (7.6)	17.6 (8.1)	11.2* (8.6)	17.6 (8.3)	10.4* (7.7)	18.4 (8.3)	13.4* (8.3)
Word Recall LR	0.59 (0.3)	0.64 (0.3)	0.43* (0.3)	0.64 (0.3)	0.46* (0.3)	0.64 (0.3)	0.44* (0.3)	0.67 (0.3)	0.52* (0.3)
Word Recall RLS	2.9 (1.4)	2.9 (1.4)	2.6 (1.4)	2.9 (1.4)	2.6 (1.4)	2.9 (1.4)	2.6 (1.4)	3.0 (1.4)	2.7 (1.4)
Word Recall LOT	4.6 (2.6)	4.7 (2.7)	4.2 (2.5)	4.8 (2.7)	4.2 (2.5)	4.7 (2.7)	4.1 (2.5)	4.8 (2.7)	4.4 (2.6)

TOC, Temporal-Occipital Classification; STOC, Simplified Temporal-Occipital Classification; LC, Lobar Classification Scheme; Chen, Chen Classification; T–, Tau negative; T+, Tau positive; RAVLT, Rey Auditory Verbal Learning Test; LR, Learning Ratio; RLS, Raw Learning Score; LOT, Learning Over Trials. All scores are raw scores, with values being *Mean* (*SD*). *Denotes significant difference between T– and T+ groups for the respective staging scheme.

**Table 3:** Effect size and Compatibility Intervals for Tau positive and Tau negative group differences among neuropsychological variables using all four pathologic staging schemes

Variable	TOC		STOC		LC		Chen	
	*Cohen*’*s d*	*95% CI*	*Cohen*’*s d*	*95% CI*	*Cohen*’*s d*	*95% CI*	*Cohen*’*s d*	*95% CI*
Learning and Memory
RAVLT Immediate Recall	0.82	0.59 – 1.05	0.76	0.55 – 0.98	0.81	0.59 – 1.03	0.57	0.38 – 0.76
RAVLT Delayed Recall	0.73	0.50 – 0.96	0.66	0.45 – 0.88	0.74	0.52 – 0.96	0.59	0.40 – 0.77
Logical Memory Immediate Recall	0.71	0.48 – 0.94	0.77	0.55 – 0.98	0.71	0.49 – 0.94	0.51	0.33 – 0.70
Logical Memory Delayed Recall	0.78	0.55 – 1.01	0.86	0.64 – 1.07	0.77	0.55 – 0.99	0.62	0.43 – 0.81
Word Recall Immediate Recall	0.87	0.64 – 1.10	0.79	0.57 – 1.00	0.91	0.69 – 1.14	0.66	0.47 – 0.85
Word Recall Delayed Recall	0.97	0.74 – 1.20	0.94	0.73 – 1.16	1.01	0.78 – 1.24	0.70	0.51 – 0.88
Learning Slope
RAVLT LR	0.82	0.59 – 1.04	0.77	0.55 – 0.98	0.83	0.61 – 1.06	0.62	0.43 – 0.81
RAVLT RLS	0.71	0.48 – 0.94	0.67	0.46 – 0.89	0.75	0.53 – 0.97	0.58	0.39 – 0.76
RAVLT LOT	0.69	0.46 – 0.91	0.67	0.46 – 0.88	0.74	0.51 – 0.96	0.57	0.38 – 0.75
Word Recall LR	0.62	0.40 – 0.85	0.57	0.36 – 0.78	0.63	0.41 – 0.85	0.51	0.32 – 0.70
Word Recall RLS	0.18	–0.04 –0.40	0.22	0.01 – 0.43	0.20	–0.02 –0.42	0.17	–0.01 –0.35
Word Recall LOT	0.16	–0.07 –0.38	0.19	–0.02 –0.40	0.19	–0.03 –0.41	0.13	–0.06 –0.31

TOC, Temporal-Occipital Classification; STOC, Simplified Temporal-Occipital Classification; LC, Lobar Classification Scheme; Chen, Chen Classification; 95% *CI*, 95% *Compatibility Interval*; RAVLT, Rey Auditory Verbal Learning Test; LR, Learning Ratio; RLS, Raw Learning Score; LOT, Learning Over Trials. *Cohen*’*s d* values and 95% *CI*s reference the results of *ANCOVAs* comparing cognitive performance between T+ and T– groups for each staging scheme. Significance is denoted by lack of overlap between the upper bound of the 95% *CI* for one variable and the mean of another.

Similarly, omnibus multivariate analyses of covariance indicated that differences also existed in learning slope metrics between T+/– groups across pathologic staging schemes after controlling for age (*Wilk*’*s Lambda* = 0.84 and *Cohen*’*s d* = 0.80 for TOC, *Wilk*’*s Lambda* = 0.86 and *Cohen*’*s d* = 0.87 for STOC, *Wilk*’*s Lambda* = 0.84 and *Cohen*’*s d* = 0.89 for LC, and *Wilk*’*s Lambda* = 0.90 and *Cohen*’*s d* = 0.68 for Chen). Group differences existed across staging schemes for the RAVLT LR (Fig. 2), RAVLT RLS, RAVLT LOT, and the Word Recall LR (*p*s < 0.001). No differences were observed for the Word Recall RLS or LOT for any of the staging schemes (*p* = 0.02–0.16). For the RAVLT LR, the magnitudes of effect were large for the TOC, STOC, and LC schemes (*Cohen*’*s ds* = 0.77 to 0.83), and medium for the Chen scheme (*Cohen*’*s d* = 0.62; Table 3). Magnitudes of effect for the RAVLT RLS, RAVLT LOT, and Word Recall LR were medium, and the Word Recall RLS and Word Recall LOT magnitudes were small. When examining 95% *CI*s, the Chen scheme failed to overlap with the midpoint of the other schemes for RAVLT LR (but not for the RLS or LOT learning slopes), suggesting that the magnitude of effect for the Chen scheme was smaller than the TOC and LC schemes for the RAVLT LR.

### ROC-AUC analyses

Tables 4 and 5 display the ROC-AUC values for both the memory and learning slope measures when differentiating individuals between the T+ and T– groups for the TOC, STOC, LC, and Chen pathologic staging schemes. For the traditional learning and memory subtests, fair AUC values were observed for the TOC, STOC, and LC schemes (0.714 to 0.792; 95% *CIs* 0.655 to 0.844), though the AUC values for the Chen scheme were poor (0.645 to 0.687; 95% *CIs* 0.595 to 0.735; Table 4). The same pattern was observed for all learning slope scores except Word Recall RLS and Word Recall LOT, which both failed to discern between biomarker groups across all pathologic staging schemes (AUCs = 0.538 to 0.561; 95% *CIs* 0.486 to 0.623; Table 5). These differences in AUC values between Chen and the other schemes were in general significant based on 95% *CIs* (for all variables except Word Recall RLS and LOT). No differences existed between TOC, STOC, and LC schemes.

**Table 4:** Receiver operating characteristic area under curve, cut scores, and sensitivity/specificity when differentiating Tau negative from Tau positive biomarker groups for learning and memory variables using the TOC, STOC, LC, and Chen classification schemes

Variable	AUC	95% CI	Cut score	Sensitivity	Specificity
RAVLT Immediate Recall
TOC	0.771	0.717 – 0.826	≤33.5	0.608	0.800
STOC	0.734	0.680 – 0.789	≤33.5	0.533	0.801
LC	0.753	0.699 – 0.807	≤33.5	0.562	0.795
Chen	0.671	0.622 – 0.721	≤33.5	0.393	0.815
RAVLT Delayed Recall
TOC	0.758	0.705 – 0.811	≤2.5	0.598	0.814
STOC	0.719	0.663 – 0.774	≤2.5	0.567	0.831
LC	0.745	0.692 – 0.798	≤2.5	0.590	0.821
Chen	0.676	0.627 – 0.725	≤2.5	0.420	0.867
Logical Memory Immediate Recall
TOC	0.726	0.667 – 0.785	≤10.5	0.649	0.708
STOC	0.726	0.672 – 0.780	≤10.5	0.633	0.727
LC	0.714	0.655 – 0.774	≤10.5	0.629	0.710
Chen	0.645	0.595 – 0.695	≤10.5	0.478	0.738
Logical Memory Delayed Recall
TOC	0.750	0.691 – 0.808	≤9.5	0.753	0.647
STOC	0.752	0.699 – 0.804	≤9.5	0.725	0.665
LC	0.737	0.679 – 0.794	≤9.5	0.714	0.645
Chen	0.676	0.627 – 0.725	≤9.5	0.567	0.687
Word Recall Immediate Recall
TOC	0.772	0.719 – 0.825	≤17.5	0.752	0.690
STOC	0.734	0.675 – 0.784	≤17.5	0.757	0.623
LC	0.768	0.714 – 0.822	≤17.5	0.758	0.682
Chen	0.687	0.639 – 0.735	≤17.5	0.784	0.476
Word Recall Delayed Recall
TOC	0.792	0.741 – 0.844	≤3.5	0.890	0.570
STOC	0.761	0.708 – 0.814	≤3.5	0.903	0.525
LC	0.784	0.733 – 0.836	≤3.5	0.899	0.570
Chen	0.686	0.638 – 0.734	≤3.5	0.932	0.357

TOC = Temporal-Occipital Classification, STOC = Simplified Temporal-Occipital Classification, LC = Lobar Classification Scheme, Chen = Chen Classification, AUC = *Area Under the Curve*, *95% CI* = 95% *Compatibility Interval*, RAVLT = Rey Auditory Verbal Learning Test.

**Table 5:** Receiver operating characteristic area under curve, cut scores, and sensitivity/specificity when differentiating Tau negative from Tau positive biomarker groups for learning slope variables using the TOC, STOC, LC, and Chen classification schemes

Variable	AUC	95% CI	Cut score	Sensitivity	Specificity
RAVLT LR
TOC	0.770	0.718 – 0.823	≤0.4495	0.690	0.779
STOC	0.732	0.676 – 0.787	≤0.4495	0.623	0.785
LC	0.759	0.705 – 0.814	≤0.4495	0.673	0.783
Chen	0.680	0.632 – 0.729	≤0.4495	0.467	0.817
RAVLT RLS
TOC	0.739	0.685 – 0.794	≤5.5	0.760	0.616
STOC	0.709	0.653 – 0.765	≤5.5	0.689	0.615
LC	0.738	0.683 – 0.793	≤5.5	0.738	0.617
Chen	0.664	0.615 – 0.714	≤5.5	0.590	0.655
RAVLT LOT
TOC	0.737	0.683 – 0.790	≤17.5	0.850	0.500
STOC	0.714	0.659 – 0.768	≤17.5	0.787	0.500
LC	0.739	0.686 – 0.792	≤17.5	0.832	0.501
Chen	0.663	0.614 – 0.712	≤17.5	0.696	0.540
Word Recall LR
TOC	0.721	0.666 – 0.777	≤0.5857	0.740	0.610
STOC	0.687	0.632 – 0.742	≤0.5857	0.689	0.615
LC	0.713	0.656 – 0.769	≤0.5857	0.738	0.617
Chen	0.654	0.604 – 0.703	≤0.5857	0.586	0.651
Word Recall RLS
TOC	0.555	0.492 – 0.619	≤1.5	0.210	0.865
STOC	0.561	0.504 – 0.619	≤1.5	0.180	0.859
LC	0.561	0.499 – 0.623	≤1.5	0.150	0.893
Chen	0.545	0.493 – 0.598	≤1.5	0.172	0.868
Word Recall LOT
TOC	0.552	0.488 – 0.615	≤7.5	0.940	0.152
STOC	0.557	0.500 – 0.614	≤7.5	0.951	0.162
LC	0.561	0.501 – 0.622	≤7.5	0.944	0.155
Chen	0.538	0.486 – 0.591	≤7.5	0.903	0.166

TOC, Temporal-Occipital Classification; STOC, Simplified Temporal-Occipital Classification; LC, Lobar Classification Scheme; Chen, Chen Classification; AUC, *Area Under the Curve*; 95% CI, 95% *Compatibility Interval*; RAVLT, Rey Auditory Verbal Learning Test; LR, Learning Ratio; RLS, Raw Learning Score; LOT, Learning Over Trials.

Additionally, we derived cut scores for the learning and memory scores, and learning slope metrics, to produce the highest balance of sensitivity and specificity for each scheme. In circumstances where different cut scores were present across schemes for a cognitive variable, we selected the cut score that displayed the most agreement across schemes. As can be seen in Tables 4 and 5, for a given cut score for a measure, the TOC, STOC, and LC schemes generated comparable sensitivity and specificity metrics, with the Chen scheme frequently generating lower sensitivity. For example, for Logical Memory Delayed Recall, a cut score of≤17.50 had a sensitivity of 0.714 to 0.753 for TOC, STOC, and LC, and a sensitivity of 0.567 for Chen. Similarly, for RAVLT LR, a cut score of≤0.4495 had a sensitivity of 0.623 to 0.673 for TOC, STOC, and LC, and a sensitivity of 0.467 for Chen. Across measures, the sensitivity and specificity data similarly tended to be stronger for the LR metrics than either RLS or LOT metrics, with comparable results between RAVLT LR metric and traditional learning and memory measures.

### Rates of tau positivity and clinical diagnostic accuracy

**Fig. 3:**
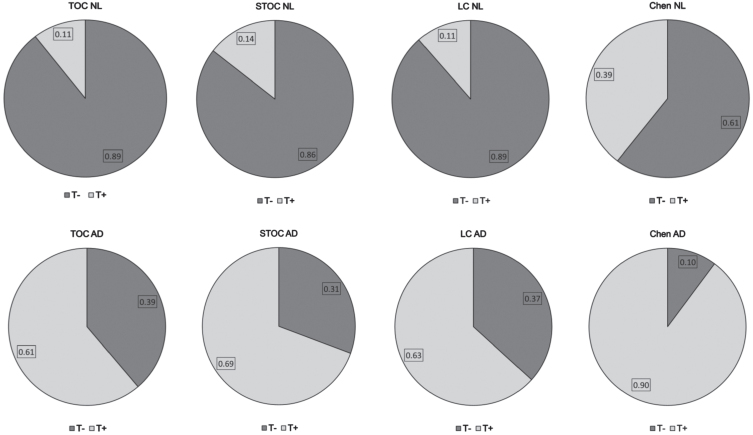
Proportions of Tau positivity and negativity among participants diagnosed as cognitively normal (NL; top row) and Alzheimer’s disease (AD; bottom row) dementia using actuarial criteria among the four pathologic staging schemes.

Finally, diagnostic composition of participants from each biomarker status group after applying actuarial methods [26, 27] were examined. The TOC, STOC, and LC schemes resulted in tau negativity for 86% to 89% of participants classified as being cognitively normal, but only 61% tau negativity for the Chen scheme (Fig. 3). Conversely, tau positivity was observed in 61% to 69% of AD dementia participants using the TOC, STOC, and LC schemes, whereas tau positivity was observed in 90% of AD dementia participants using Chen.

Further, using an actuarial diagnosis of MCI or ADNI diagnosis of AD dementia to designate cognitive impairment, we examined the diagnostic utility of the various pathologic staging schemes. Specifically, positive predictive power, which predicts how likely it is for someone to be truly clinically impaired, in case of a T+ test result, ranged from 0.644 to 0.683 for TOC, STOC, and LC schemes, but was 0.473 for the Chen scheme (Table 6). Similarly, the false positive rate, the probability of a T+ result when the true result is normal cognition, ranged from 10.5% to 14.3% for TOC, STOC, and LC schemes, whereas it was 39.5% for the Chen scheme. Finally, negative predictive power, which predicts how likely it is for someone to be truly intact, in case of a T– test result, was between 0.754 to 0.791 for allschemes.

**Table 6:** Accuracy of the pathologic staging schemes based on actuarial diagnosis

Variable	Total Sample	TOC		STOC		LC		Chen	
		T–	T+	T–	T+	T–	T+	T–	T+
Baseline Diagnosis	NL = 294	NL = 263	NL = 31	NL = 252	NL = 42	NL = 261	NL = 33	NL = 178	NL = 116
	MCI = 102	MCI = 67	MCI = 35	MCI = 60	MCI = 42	MCI = 62	MCI = 40	MCI = 42	MCI = 60
	AD = 49	AD = 19	AD = 30	AD = 15	AD = 34	AD = 18	AD = 31	AD = 5	AD = 44
Positive Predictive Power	–	0.677		0.644		0.683		0.473	
Negative Predictive Power	–	0.754		0.771		0.765		0.791	
False Positive Rate	–	10.5%		14.3%		11.2%		39.5%	

TOC, Temporal-Occipital Classification; STOC, Simplified Temporal-Occipital Classification; LC, Lobar Classification Scheme; Chen, Chen Classification; T–, Tau negative; T+, Tau positive; NL, Normal Cognition; MCI, Mild Cognitive Impairment; AD, Alzheimer’s Disease. Values for the Baseline Diagnosis variable reflect raw frequencies.

## DISCUSSION

Our results reflect the first attempt to examine criterion validity of both the Schwarz (TOC, STOC, and LC) and Chen tau topographical pathologic staging schemes, as developed from ^18^F-Flortaucipir following ADNI protocols [15, 22–24]. In Schwarz and colleagues’ [10] original manuscript, the authors developed the TOC, STOC, and LC staging schemes in 35 scans and then compared results to amyloid-status in 98 participants from ADNI-2 [23]. They observed high concordance (90% to 94%) between TOC, STOC, and LC schemes, with 82% to 96% of participants classified as T+ also being Aβ positive; conversely, 35 to 41% participants classified as T– were Aβ positive. Our findings with an expanded dataset (*n* = 465) from ADNI appeared comparable. Concordance across Schwarz schemes was 88% to 95%, 86% to 87% of T+ participants were Aβ positive, and 43% to 45% of T– participants were Aβ positive (Table 1). However, until now Schwarz schemes have not been evaluated in relation to clinical outcomes. The current study investigated this by 1) comparing learning and memory measures, and learning slope metrics, and 2) examining diagnostic accuracy between T+/T– groups across all three Schwarz pathologic staging schemes (TOC, STOC, and LC). Our results suggest that traditional memory measures were significantly different for T+/T– groups across all pathologic staging schemes, with large magnitudes of effect. Differences were also observed for all pathologic staging schemes for learning slopes derived from RAVLT (LR, RLS, and LOT) and Word Recall (LR), with similar magnitudes of effect. In all cases, tau positivity was associated with worse cognitive performance (Tables 2 and 3). Relatedly, memory and learning slope measures were nearly all categorized as having “fair” sensitivity at classifying tau biomarker status using the TOC, STOC, and LC schemes (Tables 4 and 5). Together, these results are consistent with studies relating RAVLT, Logical Memory, and ADAS-Cog to AD biomarkers of Aβ and tau [49–52], as well as some growing literature on the relationship between learning slopes and both Aβ and tau status [18, 52]. Overall, these results provide converging evidence that the pathologic staging schemes as developed by Schwarz and colleagues are sensitive to cognitive outcomes.

When attempting to discriminate between utility of the three Schwarz schemes relative to cognitive and clinical outcomes, our results suggest high overlap. For example, the proportion of the sample classified as T+ was highly similar (ranging from 73% to 78%; Table 1), the magnitudes of effect for differences between memory and learning slope measures were comparable (mean *Cohen*’*s d* value ranging from 0.80 to 0.83; Table 3), and both the AUC and sensitivity/specificity values for a given cognitive cut point were consistent (e.g. for RAVLT Total Recall, AUC = 0.734 to 0.771, sensitivity = 0.533 to 0.608, specificity = 0.795 to 0.801; Table 4) across all three schemes. Although Table 6 suggests a slightly higher false positive rate for STOC (14.3%) relative to the others (10.5% to 11.2%), this difference is not clinically meaningful. Consequently, there appears no difference in clinical outcomes between these three schemes, despite their varying ROI specificity and incorporation of temporal tau accumulation. However, Schwarz and colleagues previously suggested “more robust performance in terms of fewer unclassified scans and increased test-rest reproducibility of assigned stage” ([10]; pp. 221) by the schemes with less ROIs and less complicated decision rules (i.e., STOC and LC). Anecdotally, our experience in applying these staging procedures corresponds to the authors’ observation, such that the more complicated scoring rules like the TOC scheme resulted in a slightly higher frequency of cases that did not “fit” the disease-progression models. This differential fit was associated with a small number of participants possessing high overall tau deposition, but having deposition patterns that did not map onto the anticipated progression of AD tau deposition spread (starting in the transentorhinal cortex and ending in primary visual cortex) [8, 9]. Consequently, given the strength of the results and their ease of use, the LC and STOC schemes may confer advantages to the more complicated TOC scheme.

In addition to the Schwarz staging schemes, the current study also examined the validity of Chen and colleagues’ [12] pathologic staging scheme. We found that dichotomization of tau using the Chen scheme resulted in T+ classified individuals having significantly worse cognitive performances than their T– peers; in particular, T+ participants performed on average 0.61 *SD* lower across memory and 0.57 *SD* lower than T– individuals across most learning slope scores (Table 3). This is consistent with the results of Chen and colleagues’ original manuscript, where comparison to longitudinal cognitive performance indicated worsening rates of cognitive decline and clinical progression.

While these findings offer support for the Chen scheme, several other observed results were not as positive. First, use of AUC-ROC analyses revealed that cognitive measures were categorized as having “poor” sensitivity at classifying tau biomarker status using the Chen scheme (Tables 4 and 5). Second, across both multivariate analysis of covariance and AUC-ROC analyses, examination of 95% *CI*s suggests that the magnitudes of effect were consistently smaller for the Chen scheme than the TOC, STOC, or LC schemes (Tables 2–5). Third, while negative predictive power was comparable across staging schemes, positive predictive power—which predicts how likely it is in the case of a T+ test result for someone to be truly cognitively impaired using an actuarial diagnosis—was lower in the Chen scheme than in the others (Table 6). This lower finding is consistent with the rate of false positives being approximately four times higher for Chen than the other schemes (40.1% versus 10.4% to 14.5%), and the fact that the Chen scheme resulted in a 49% positivity rate (relative to a mean positivity rate of 25% across TOC, STOC, and LC) for the same participants. Of note, there is no standard “cutoff” of positive predictive or negative predictive power values to denote “good” or “bad” test characteristics, but instead the importance of true positive versus true negative (or false positive versus false negative) determinations must be balanced for the specific purpose [53]. In our current circumstance, with all negative predictive power values being relatively equal, the lower positive predictive power values for Chen (and higher false positive rates) relative to the TOC, STOC, or LC schemes was subsequently viewed as suboptimal. It is possible that we may have introduced bias into the diagnostic accuracy analyses by using actuarial diagnosis as our clinical outcome anchor. However, we observed that our cognitively impaired participants (i.e., those classified with AD dementia or MCI) performed significantly worse across cognitive measures, possessed smaller bilateral hippocampal volumes, and had higher rates of Aβ positivity than those in the cognitively normal group. This provides higher confidence to assert that the disparity between T+ rates for the Chen and Schwarz schemes is related to Chen’s elevated sensitivity and false positive rate, relative to Schwarz’s increased specificity.

**Fig. 4:**
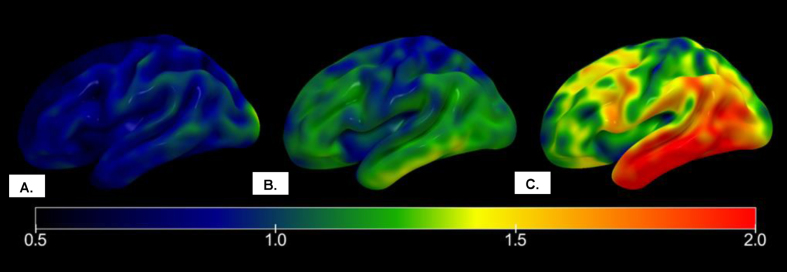
Mean right hemispheric ^18^F-Flortaucipir SUVR maps of participants with normal cognition, mild cognitive impairment, and Alzheimer’s disease in ADNI. A) Tau maps for a participant classified as T– by both TOC and Chen pathologic staging schemes. B) Tau map for participant classified as T+ by Chen staging scheme but T– by TOC staging scheme. C) Tau map for participant classified as T+ by both TOC and Chen staging schemes. SUVR, standardized uptake value ratio; ADNI, Alzheimer’s Disease Neuroimaging Initiative.

As a result, the Chen scheme may not have as high of utility for classifying tau pathology as the Schwarz schemes. When considering that the two sets of schemes generally incorporated comparable regions of interest with similar staging processes, a possibility for these differential findings between groups may be the choice of cut point for the Chen scheme. Specifically, the authors observed that because the earliest cognitive decline was detected by the memory composite in stage 1 of their staging process, “the SUVR threshold in Braak I/II ROI classifying stage 0 and stage 1 might be considered as the cutoff of tau biomarker to define Alzheimer’s disease” [12]. However, these higher false positive rates, relative to the TOC, STOC, or LC schemes, suggest that this staging cutoff may have been too liberal. When applying these stages to neuroanatomical correlates, the suggested cutoff for tau positivity at Chen stage 1 (range 0 to 4) equates to “a dominating tau elevation in medial temporal regions (Braak I/II ROIs)” [12]. Conversely, the suggested cutoff for tau positivity at TOC stage 4 (range 0 to 6) equates to tau accumulation in the hippocampus, trans-entorhinal cortex, fusiform gyrus, middle temporal gyrus, and extra-striate visual cortex (though hippocampal or extra-striate visual sparing was possible). Consequently, a cutoff of 1 for Chen involves notably less tau accumulation in AD-specific neuroanatomical regions than a cutoff of 4 for TOC (see Fig. 4), which may explain the discrepancy in T+ rates between the schemes. While it is tempting to suggest that a more conservative cutoff for tau positivity for the Chen scheme—from stage 1 to stage 2—would lead to findings more in line with the Schwarz schemes, 169 of the current sample of 465 participants were classified as Chen stage 1. This means that transitioning those participants from being T+(as in the current decision tree) to T– would result in a drop of the T+ rate of from 49% to 13%, which may be too extreme of a compensation (leading to positive predictive power improving from 0.459 to 0.857, and false positivity rate declining from 40.1% to 2.7%) at the expense of sensitivity. Adjustment of Chen’s SUVR thresholds for stages 1 or 2 may be more advisable, and future research is required to properly consider the strategy of modifying thresholds or cutoff scores for the Chen scheme to optimize its utility when applied to clinical outcomes.

Finally, although the focus of this manuscript was on the tau topographic staging schemes, it should not be overlooked that performance by select learning slope metrics performed comparably to more established memory measures. In particular, the LR metric derived from the RAVLT displayed similar magnitudes of effect (Table 3) and AUC values (Table 5) relative to memory measures from the RAVLT, Logical Memory, and the ADAS-Cog. These findings correspond with the limited research investigating learning slopes and AD biomarkers of Aβ [18] and tau [52]. As the LR metric from the RAVLT appeared to outperform that derived from Word Recall of the ADAS-Cog, it is possible that the greater number of trials (5 versus 3) and words per trial (15 versus 10) improved the sensitivity for the former, though future research is needed to thoroughly investigate the effect of trial number and length on learning slope performance.

Our current study is not without limitations. First, these results are unique to the Schwarz and Chen tau pathologic staging schemes as applied to ^18^F-Flortaucipir PET imaging according to ADNI protocols, using SUVR thresholds as developed by the original authors. Appropriateness of other tau staging schemes or decision rules cannot be inferred from these results, particularly as they relate to different imaging radioligands or scanning protocols. Second, our use of a sample from ADNI has resulted in a disproportionately large number of highly-educated non-Hispanic white adults, who have met stringent exclusion criteria specific to ADNI and industry-sponsored clinical trials. Future development of tau staging schemes outside of the ANDI framework (e.g., [54]) will be necessary to broaden generalizability of these findings. Third, our use of ADNI data led to the incorporation of neuropsychological test measures into the study that have been modified specially for ADNI (e.g., Logical Memory only includes “Story A”). Relatedly, the original Chen et al. [12] manuscript included the ADNI-Memory composite [55] in its validation study, which incorporates some – but not all – of the memory measures used in the present study. Although the overlap may have led to overly similar results between studies, the Chen study focused on stages of tau pathology (range 0 to 4) whereas ours examined overall tau positivity/negativity. Our contrasting findings for the Chen scheme, particularly in relation to the Schwarz schemes, support that our results were not confounded by the memory measures used. Fourth, it could be questioned, however, that the high concordance between the TOC, STOC, and LC schemes may be due to shared method variance, given that they were all developed by Schwarz and colleagues on the same cohort. While this likely contributes to some of the differences in concordance between these schemes and the Chen scheme, the Chen scheme developed pathologic staging dichotomization based on *T*+ corresponding to PET positivity anywhere in the transentorhinal, limbic, and neocortical regions, whereas the TOC, STOC, and LC schemes possessed a higher threshold for *T*+(scans with regional positivity restricted to medial temporal regions [transentorhinal cortex, hippocampus, and fusiform gyrus] were considered T–). As a result, we do not feel that this shared method variance explains the entirety of the discrepancies in the false positive rates between TOC/STOC/LC and Chen schemes. Fifth, Immediate Recall and Delayed Recall from the ADAS-Cog Word Recall (Questions 1 and 4 of the ADAS-Cog) were calculated in a different manner than recommended by test developers. Although this was conducted for comparability with the RAVLT and Logical Memory (i.e., higher scores meant better memory performance) and has been used previously [52], this scoring is not universally agreed-upon. Fifth, as alluded to previously our use of the actuarial diagnosis [26, 27] may have introduced bias into the calculation of false positive rates, as patients may have been tau positive years prior to the manifestation of clinical symptoms. However, we hope to have shown, based on cognitive impairment, hippocampal atrophy, and Aβ deposition, that our cognitively impaired sample had probable AD pathology. Relatedly, the use of an FAQ score≥6 for a cutoff between MCI and normal cognition in the actuarial criteria may have resulted in some participants with objective functional impairment being classified as MCI; this is because it is unknown whether our participants with FAQ scores of 6 or greater had mild functional difficulty in multiple domains, or dependence in two domains. While the actuarial criteria were only applied to participants in this study with ADNI diagnoses of MCI or normal cognition—not dementia due to AD—it remains a possibility that some percentage of MCI participants in our sample had functional impairment despite MCI classification using both actuarial and ADNI diagnostic criteria. Sixth, the consideration of the Research Framework in this study does not constitute endorsement of using a pathology-only criteria for classification or diagnosis of AD. Conversely, the authors encourage continued utility for neuropsychological testing in the clinical diagnosis of dementia due to AD. Finally, a question could be raised about the overall appropriateness of advocating for dichotomization of T in the “ATN” model given the relative novelty of tau-PET imaging. This manuscript does not propose to be a definitive opinion on the appropriateness of dichotomization of tau-PET results, however the authors sought to understand the validity of current methods given the tendency for researchers to dichotomize them as a result of the needs of the “ATN” Research Framework. It is expected that the maintenance of tau SUVR values as continuous variables will retain importance, particularly in both clinical and research settings. Additional consideration of the appropriateness of tau-PET dichotomization is warranted.

Limitations withstanding, the current study appears to provide evidence of criterion validity for these different tau pathologic staging schemes, when examined in the context of traditional learning and memory measures, learning slope metrics, and actuarial diagnoses. Although results were comparable between the TOC, STOC, and LC schemes of Schwarz, ease of use and better data fit preferred the STOC and LC schemes. While some evidence was supportive for the Chen scheme, validity lagged behind the other schemes, likely due to elevated false positive rates. Tau PET staging schemes appear to be valuable for AD diagnosis, tracking, and screening for clinical trials. The validation of these schemes subsequently provides support for their use as options for tau pathologic dichotomization (T+ versus T–), which will advance the use of the NIA-AA Research Framework (“ATN” model) when using tau-PET techniques. Future research should consider other staging schemes and validation with other outcome benchmarks.

## Supplementary Material

Supplementary Material

## Data Availability

The data supporting the findings of this study are available on request from the corresponding author. The data are also publicly available through ADNI.
