# Supplementary material for: Criterion Validation of Tau PET Staging Schemes in Relation to Cognitive Outcomes
Source: J Alzheimers Dis. 2023 Oct 24;96(1):197–214. doi: 10.3233/JAD-230512 (PMC10825758; doi:10.3233/JAD-230512)
Supplement: Supplementary Material [file jad-96-jad230512-s001.pdf]

Supplementary Material

Criterion Validation of Tau PET Staging Schemes in Relation to Cognitive Outcomes

Supplementary Table 1. Abbreviations used in the current study.

Abbreviation	Definition
**AD**	Alzheimer's disease
**Aβ**	Amyloid-β
**PET**	Positron Emission Tomography
**NIA-AA**	National Institute of Aging – Alzheimer's Association
**ATN**	Amyloid-Tau-Neurodegeneration
**TOC**	Temporal-Occipital Classification
**STOC**	Simplified Temporal-Occipital Classification
**ROI**	Regions of Interest
**LC**	Lobar Classification
**Chen**	Chen Classification
**ADNI**	Alzheimer's Disease Neuroimaging Initiative
**SUVR**	Standardized Uptake Value Ratio
**MCI**	Mild Cognitive Impairment
**ADAS-Cog**	Alzheimer's Disease Assessment Scale – Cognitive Subscale
**RAVLT**	Rey Auditory Verbal Learning Test
**RLS**	Raw Learning Score
**LR**	Learning Ratio
**LOT**	Learning Over Trials
**AUC-ROC**	Receiver Operating Characteristic Area Under the Curve
**CI**	Compatibility Interval

Explanation of SUVR cutoffs

The following is a description of approaches from Schwarz et al. [1] and Chen et al. [2] to derive SUVR cutoffs in their original studies.

TOC, STOC, LC schemes. Schwarz et al., 2018 [1]

From the Supplement of Schwarz et al., 2018 (pp. 7): "*We selected threshold SUVR values for each classification scheme in a way that allowed ROI-specific thresholds (accounting for different background signal characteristics in different brain regions) but maintained a consistent ROI-average SUVR threshold (of approximately 1.28). To achieve this, the threshold values for TOC were calculated, for each ROI, as 2.5 standard deviations above the mean value from a young, cognitively normal, reference group. To achieve comparable absolute threshold levels, the threshold values for STOC and LC were calculated as 3 standard deviations above the mean.*"

Chen scheme. Chen et al., 2021 [2]

According to the authors, SUVR values were derived by Schöll et al. [3] and Maass et al. [4].

From Chen et al., 2021 (pp. 2). As described, "*In the original [Scholl and Maass] work, a conditional inference tree was employed to classify subjects with regard to their clinical diagnosis (i.e., young controls, older cognitively normal controls, Alzheimer's disease). An SUVr threshold in Braak V/VI ROI was first derived with the whole sample entering the model. The participants above this threshold were classified as the highest stage. After the removal of those participants, the staging and threshold-deriving procedure continued with the next Braak ROI (III/IV). Continuing this approach, three thresholds could be obtained and those reaching no threshold were defined as the lowest stage. More details in the generation of the thresholds could be found in their work.*"

REFERENCES

[1] Schwarz AJ, Shcherbinin S, Slieker LJ, Risacher SL, Charil A, Irizarry MC, Fleisher AS, Southekal S, Joshi AD, Devous MD, Sr., Miller BB, Saykin AJ (2018) Topographic staging of tau positron emission tomography images. *Alzheimers Dement (Amst)* **10**, 221-231.

[2] Chen S-D, Lu J-Y, Li H-Q, Yang Y-X, Jiang J-H, Cui M, Zuo C-T, Tan L, Dong Q, Yu J-T, et al. (2021) Staging tau pathology with tau PET in Alzheimer's disease: a longitudinal study. *Transl Psychiatry* **11**, 483.

[3] Schöll M, Lockhart SN, Schonhaut DR, O'Neil JP, Janabi M, Ossenkoppele R, Baker SL, Vogel JW, Faria J, Schwimmer HD, Rabinovici GD, Jagust WJ (2016) PET imaging of tau deposition in the aging human brain. *Neuron* **89**, 971–982.

[4] Maass A, Landau S, Baker SL, Horng A, Lockhart SN, La Joie R, Rabinovici GD, Jagust WJ; Alzheimer's Disease Neuroimaging Initiative (2017) Comparison of multiple tau-PET measures as biomarkers in aging and Alzheimer's disease. *Neuroimage* **157**, 448-463.

Supplementary Table 2. Comparison of cognitive and functional abilities, and amyloid status, between actuarial diagnostic groups

Variable	Cognitively Normal	MCI	AD
**MOCA** [1,2,3]	25.90 (2.7)	22.56 (3.7)	16.87 (4.8)
**CDR-SB** [1,2,3]	0.23 (0.5)	1.59 (1.3)	4.35 (1.8)
**ADAS-Cog** [1,2,3]	13.04 (4.2)	20.57 (6.3)	32.61 (6.3)
**RAVLT Immediate Recall** [1,2,3]	46.05 (10.0)	34.43 (10.2)	22.78 (7.0)
**RAVLT Delayed Recall** [1,2,3]	7.80 (4.2)	3.58 (3.4)	0.55 (1.9)
**Hippocampal Volume** [1,2,3]	3816.54 (392.64)	3596.92 (556.61)	2968.89 (516.13)
**Amyloid Positivity (%)** [1,2,3]	45%	65%	90%

MCI, mild cognitive impairment; AD, Alzheimer's disease; MoCA, Montreal Cognitive Assessment; CDR-SB, Clinical Dementia Rating Scale – Sum of Boxes; ADAS-Cog, Alzheimer's Disease Assessment Scale – Cognitive Subscale; RAVLT, Rey Auditory Verbal Learning Test. All scores are raw scores, and all values are *Mean* (*SD*) unless listed otherwise. Hippocampal volume is bilateral volume in mm^3.

[1] Denotes significant difference between Cognitively Normal and MCI groups, $p<0.001$.

[2] Denotes significant difference between Cognitively Normal and AD groups, $p<0.001$.

[3] Denotes significant difference between Cognitively MCI AD groups, $p<0.001$.
